# Supplementary material for: Mapping the molecular and structural specialization of the skin basement membrane for inter-tissue interactions
Source: Nat Commun. 2021 May 10;12:2577. doi: 10.1038/s41467-021-22881-y (PMC8110968; doi:10.1038/s41467-021-22881-y)
Supplement: Supplementary file 1 — Supplementary Information [file 41467_2021_22881_MOESM1_ESM.pdf]

Supplementary Information

Mapping the molecular and structural specialization of the skin basement membrane for inter-tissue interactions

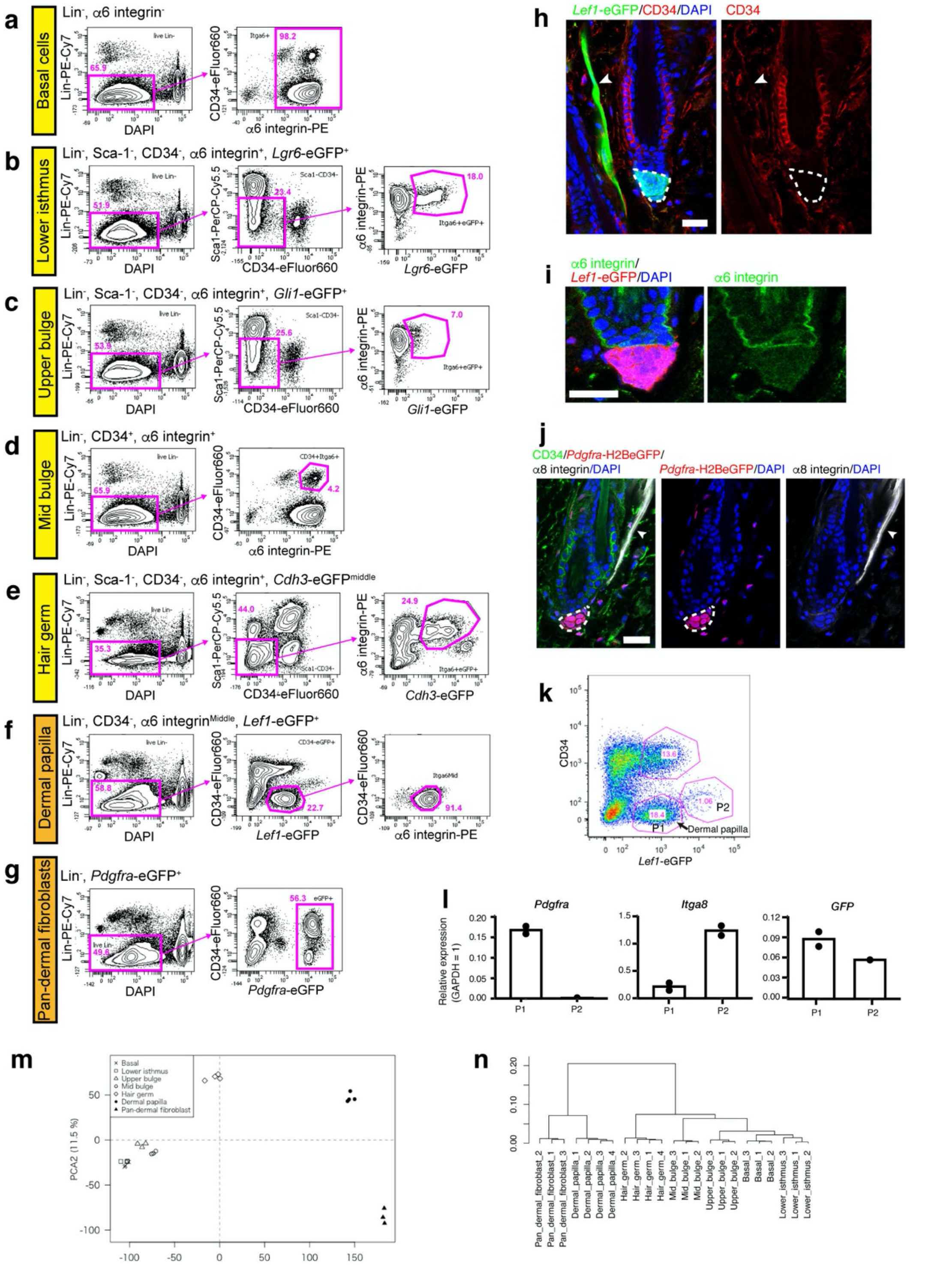

Tsutsui et al., Figure S1

Supplementary Fig. 1 Isolation of hair follicle epithelial and dermal cell sub-populations. **a–e** FACS-based cell isolation procedures for basal epithelial cells in whole dorsal epithelium (basal cells) (a), lower isthmus (b), upper bulge (c), mid-bulge (d) and hair germ (HG) (e), according to our previous study[1]. Whole dorsal skin from 8-week-old female mice was used. Red polygons indicate sorting gates. **f, g** FACS-based cell isolation procedures for dermal papilla (DP) (f) and pan-dermal fibroblasts (g). **h** Expression pattern of CD34 in the *Lef1*-eGFP-positive cell populations. Neither DP cells (encircled by a dashed line) nor arrector pili muscles (white arrowhead) express CD34. **i** Weak expression of α6 integrin in the *Lef1*-eGFP-positive telogen DP cells. **j** Distinct marker expression in DP and arrector pili muscles. Eight-week-old adult telogen skin of *Pdgfra*-H2BeGFP mice was stained for CD34, GFP, α8 integrin and nuclei (DAPI). DP (encircled by a dashed line) cells were $CD34^{-}/PDGFRA^{+}/\alpha8\ integrin^{low}$. Arrector pili muscles were $CD34^{dim}/PDGFRA^{-}/\alpha8\ integrin^{high}$. **k** FACS gate setting for DP cell isolation. Upon Lin^{+} and dead cell depletion, single-cell suspensions of 8-week-old adult telogen dermal tissues of *Lef1*-eGFP mice were plotted based on the signal intensity of CD34 and *Lef1*-eGFP. Two gates (P1 and P2) were used to isolate $CD34^{-}$/*Lef1*-$eGFP^{middle}$ (P1) and $CD34^{middle}$/*Lef1*-$eGFP^{high}$ (P2). **l** Quantitative RT-PCR gene expression analysis of DP and arrector pili muscle marker genes in P1 and P2 cell populations in k. The P1 population showed high *Pdgfra* expression and low *Itga8* expression, consistent with the character of DP. In contrast, the P2 cell population showed no *Pdgfra* expression and high *Itga8* expression, indicating that this cell population is arrector pili muscles. *Lef1*-eGFP expression was detected in both P1 and P2 populations. Data are mean with each data point. n=2 mice. **m** Principal component analysis (PCA) of all cell populations analysed in this study. All biological replicates of the same population form close clusters, indicating high reproducibility of our method. **n** Hierarchical clustering analysis of HF cell populations. All expressed genes were used in this analysis. All biological replicates of the same population form close clusters, indicating high reproducibility of our method. Scale bars, 20 μm.

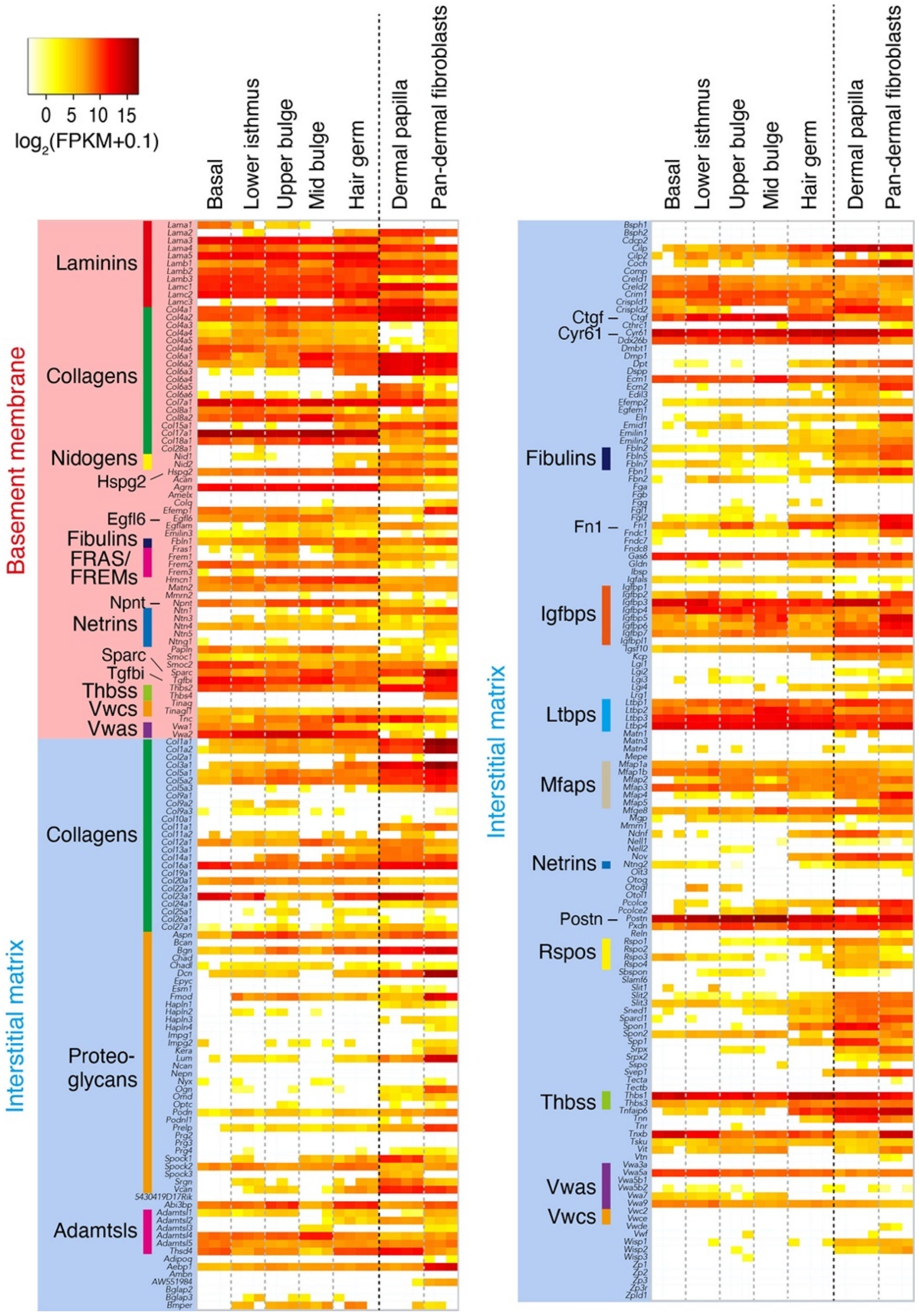

Supplementary Fig. 2 Overview of the matrisome gene expression. Heatmap representation of the expression levels of all matrisome genes. FPKM values of each biological replicate (log_2-converted) are indicated according to a heat gradient as shown at the top left corner. Matrisome genes are first categorized into basement membrane (BM) components (red zone) and interstitial matrix components (blue zone). Then, they are subdivided according to the ECM families to which they belong. Epithelial and dermal compartments are separated by a dashed line.

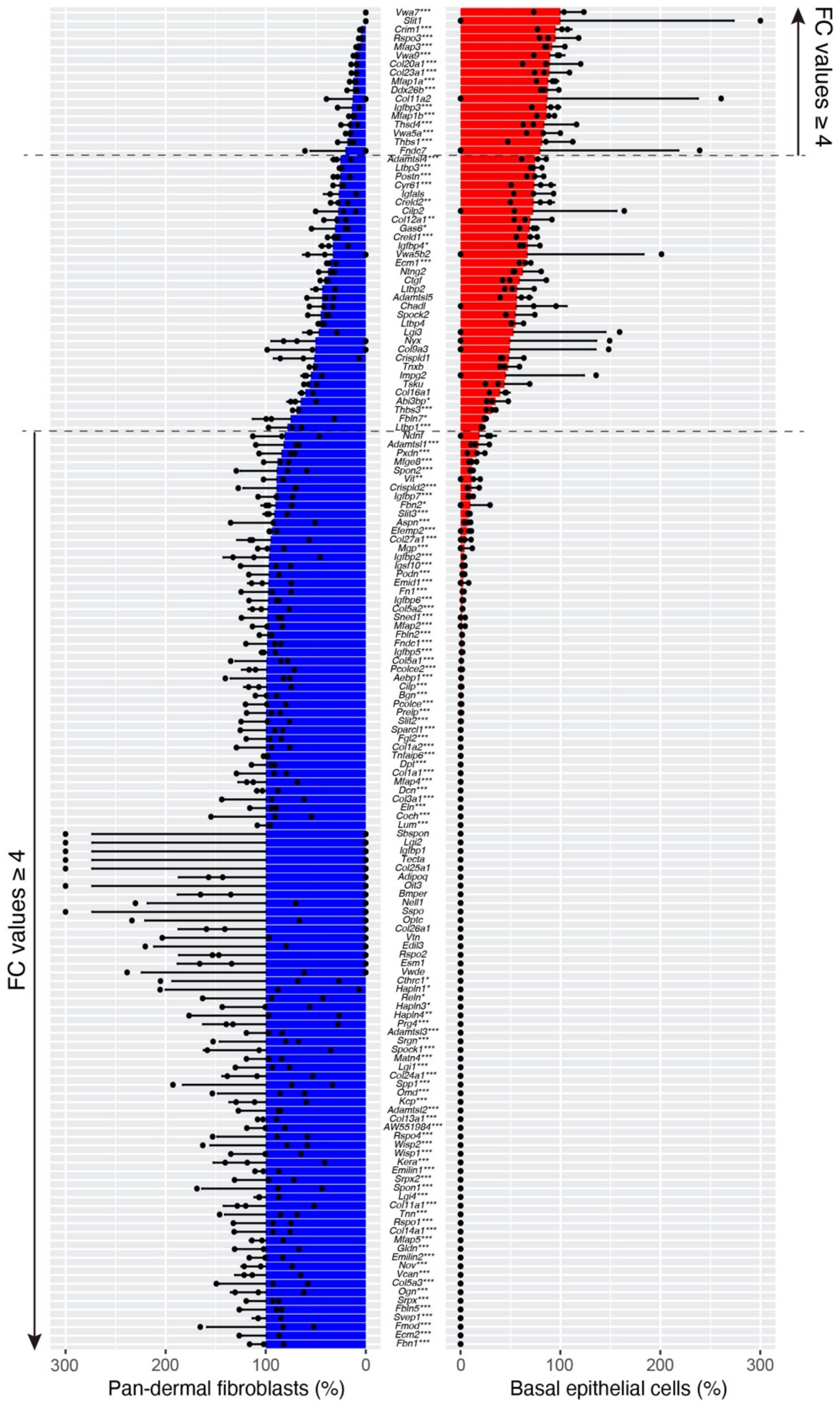

Supplementary Fig. 3 Comparison of expression levels of interstitial ECM genes between epithelial and fibroblast cells. Bar graph represents the ratio of expression of interstitial ECM genes in the basal epithelial cell population and pan-dermal fibroblast cell population. Data are mean ± SD of three biological replicates. Adjusted *p*-values from two-sided Wald test are shown: **p*<0.05; ***p*<0.01; ****p*<0.001.

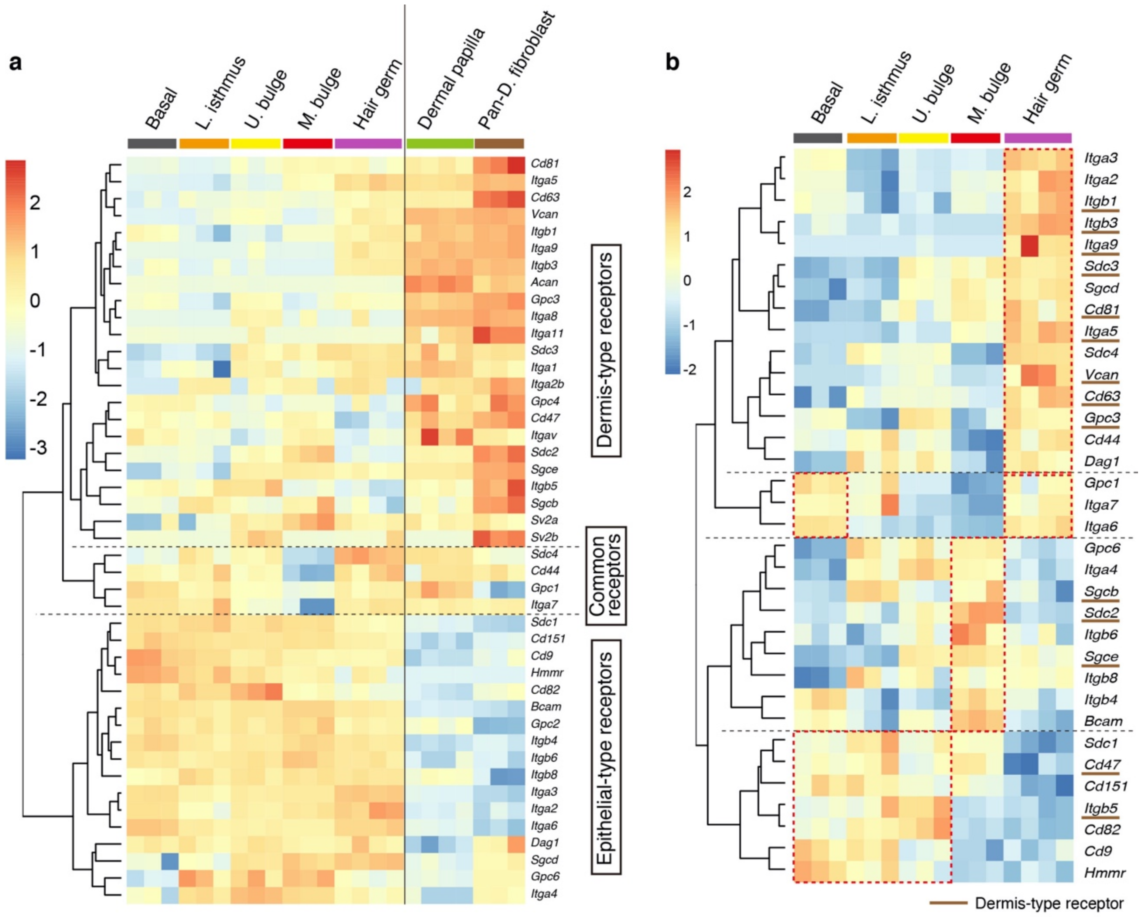

Supplementary Fig. 4 Heatmap representing the hierarchical clustering of the expression patterns of ECM receptor genes. a All epithelial and dermal cell populations were analysed. ECM receptors are sub-divided into three clusters: epithelial-type, dermis-type and universal receptors. **b** Only epithelial cell populations were analysed. ECM receptors are sub-divided into four clusters: hair germ (HG)-type, basal/HG-type, mid-bulge-type and basal/lower isthmus/upper-bulge-type receptors. Brown bars under gene names indicate dermis-type receptors identified in a.

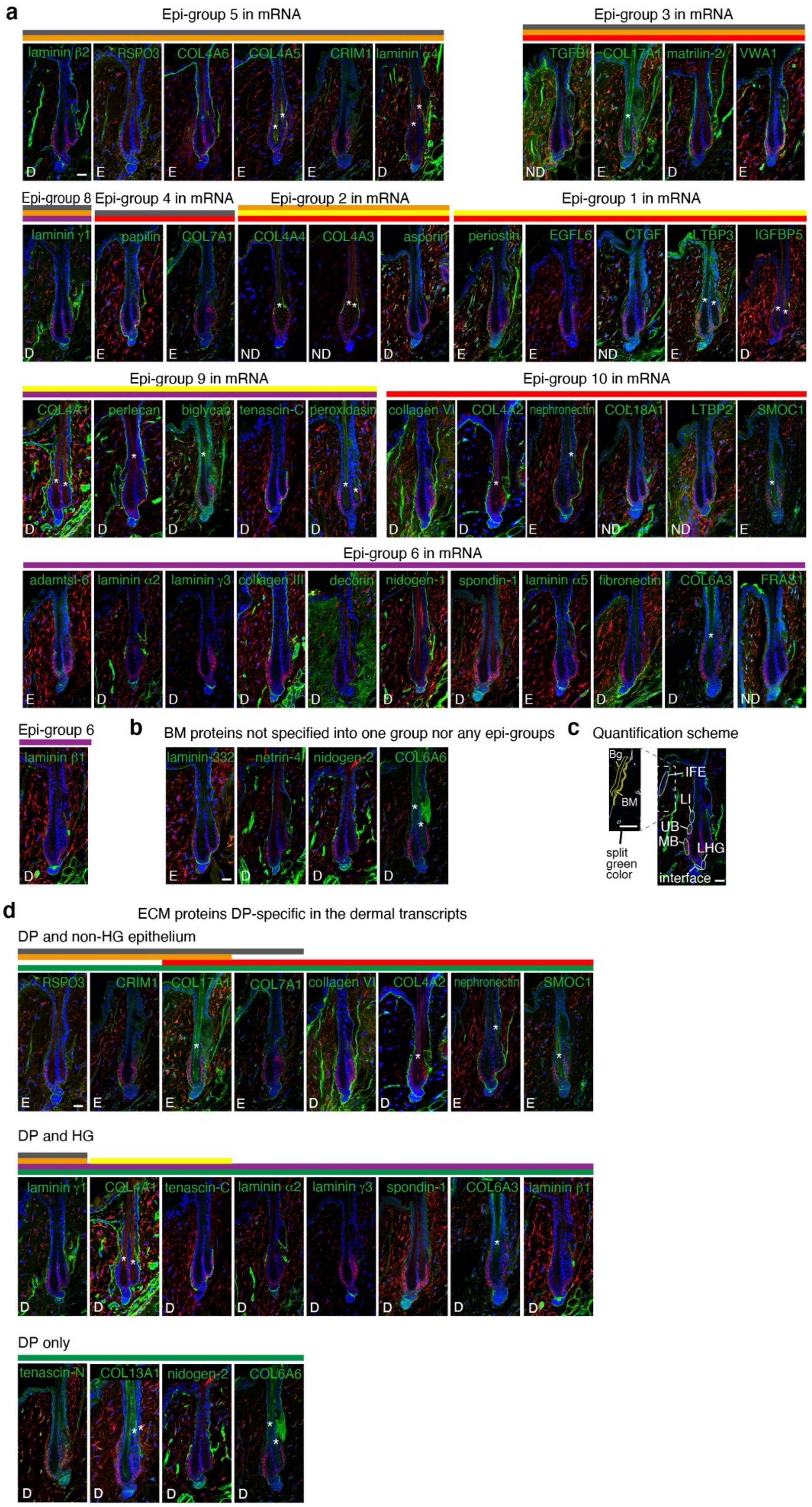
a
Epi-group 5 in mRNA
laminin β2
RSPO3
COL4A6
COL4A5
CRIM1
laminin α4
Epi-group 3 in mRNA
TGFBI
COL17A1
matrilin-2
VWA1
Epi-group 8
Epi-group 4 in mRNA
Epi-group 2 in mRNA
Epi-group 1 in mRNA
laminin γ1
papilin
COL7A1
COL4A4
COL4A3
asporin
periostin
EGFL6
CTGF
LTBP3
IGFBP5
Epi-group 9 in mRNA
Epi-group 10 in mRNA
COL4A1
perlecan
biglycan
tenascin-C
peroxidasin
collagen VI
COL4A2
nephronectin
COL18A1
LTBP2
SMOC1
Epi-group 6 in mRNA
adamtsl-6
laminin α2
laminin γ3
collagen III
decorin
nidogen-1
spondin-1
laminin α5
fibronectin
COL6A3
FRAS1
Epi-group 6
laminin β1
b
BM proteins not specified into one group nor any epi-groups
laminin-332
netrin-4
nidogen-2
COL6A6
c
Quantification scheme
Bg
BM
split green color
IFE
LI
UB
MB
LHG
interface
d
ECM proteins DP-specific in the dermal transcripts
DP and non-HG epithelium
RSPO3
CRIM1
COL17A1
COL7A1
collagen VI
COL4A2
nephronectin
SMOC1
DP and HG
laminin γ1
COL4A1
tenascin-C
laminin α2
laminin γ3
spondin-1
COL6A3
laminin β1
DP only
tenascin-N
COL13A1
nidogen-2
COL6A6

Supplementary Fig. 5 Immunolocalizations of ECM proteins in the telogen hair follicle. a Tissue localizations of protein products of ECM genes region-specifically expressed by the epithelial cell populations. Individual ECM proteins (green) were stained with CD34 (red) and DAPI (blue). Their classified epi-group numbers are indicated at the top of each panel with colour bars as shown in Fig. 3. **b** Tissue localizations of protein products of basement membrane (BM) genes that are not classified in any specific epi-group. **c** Example of protein intensity measurement. Each image was colour-split. Then average intensities of six target BM regions were measured with subtracting background signals in the adjacent epithelial regions (Bg). IFE: inter follicular epidermis, LI: lower isthmus, UB: upper bulge, MB: mid-bulge, LHG: lateral hair germ. **d** Tissue localizations of protein products of dermal papilla (DP) specific-ECM genes. These genes are divided into three groups by their expression patterns in the epithelium. This information is indicated with colour bars. Scale bar, 20 μm.

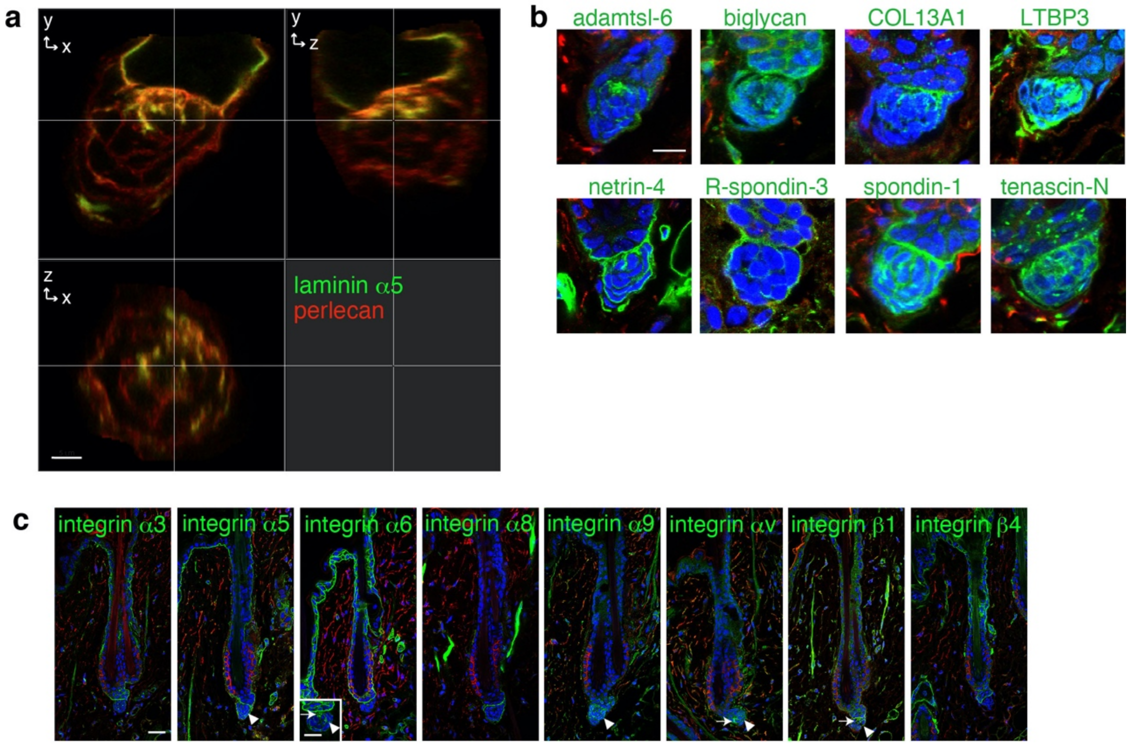

Supplementary Fig. 6 Tissue localization of ECM and ECM receptor proteins in the hook and mesh basement membranes. a Three-dimensional observation of the hook and mesh basement membranes (BMs) in Fig. 7a. **b** Localizations of ECM proteins composing hook and mesh BMs. Images are magnified views of images in Fig. S5. **c** Immunolocalizations of various integrin receptors in the dorsal telogen HFs. Integrins are shown in green with CD34 (red) and nuclear (blue) stains. White arrows and arrowheads indicate hook BM-like localizations and mesh BM-like localizations of integrins, respectively. The inset in the integrin α6 image shows the dermal papilla (DP) region of a different hair follicle. Scale bars: 5 μm (a), 10 μm (b) and 20 μm (c).

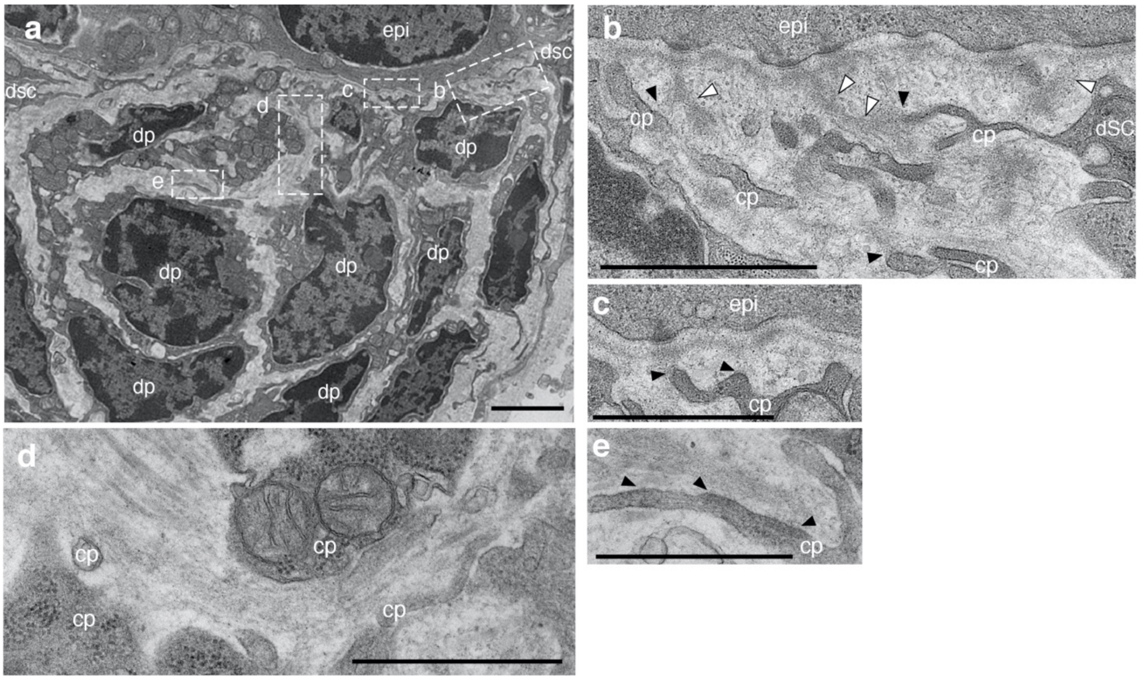

Supplementary Fig. 7 Electron microscopic images of cellular protrusions extending toward the basement membrane structures within the dermal papilla. a Transmission electron microscopy (TEM) image of the dermal papilla (DP). Dotted rectangles indicate the regions of interest magnified in (b–e). **b–e** Magnified views of characteristic ECM structures. Cellular protrusions (cp) interact with protrusions of the interface basement membrane (BM) (white arrowheads). Black filled arrowheads indicate the interaction sites. Hair follicle dermal stem cells located at the upper edge of DP extended long protrusions toward the interface between hair germ (HG) and DP and appeared to form direct contacts with these BM protrusions and the interface BM (b, c). DP cells extended protrusions toward the hook BM and appeared to form cell adhesion-like structures (d, e). Cellular protrusions have electron-dense membrane structures at cell–BM interfaces (black filled arrowheads in b, c, e). Large extracellular space at the centre of the DP is filled with thick bundles of ECMs (d). epi, epithelial HG cell; dp, DP cell; dsc, dermal stem cell; cp, cellular protrusion. Scale bars: 2 μm (a), 1 μm (b–e).

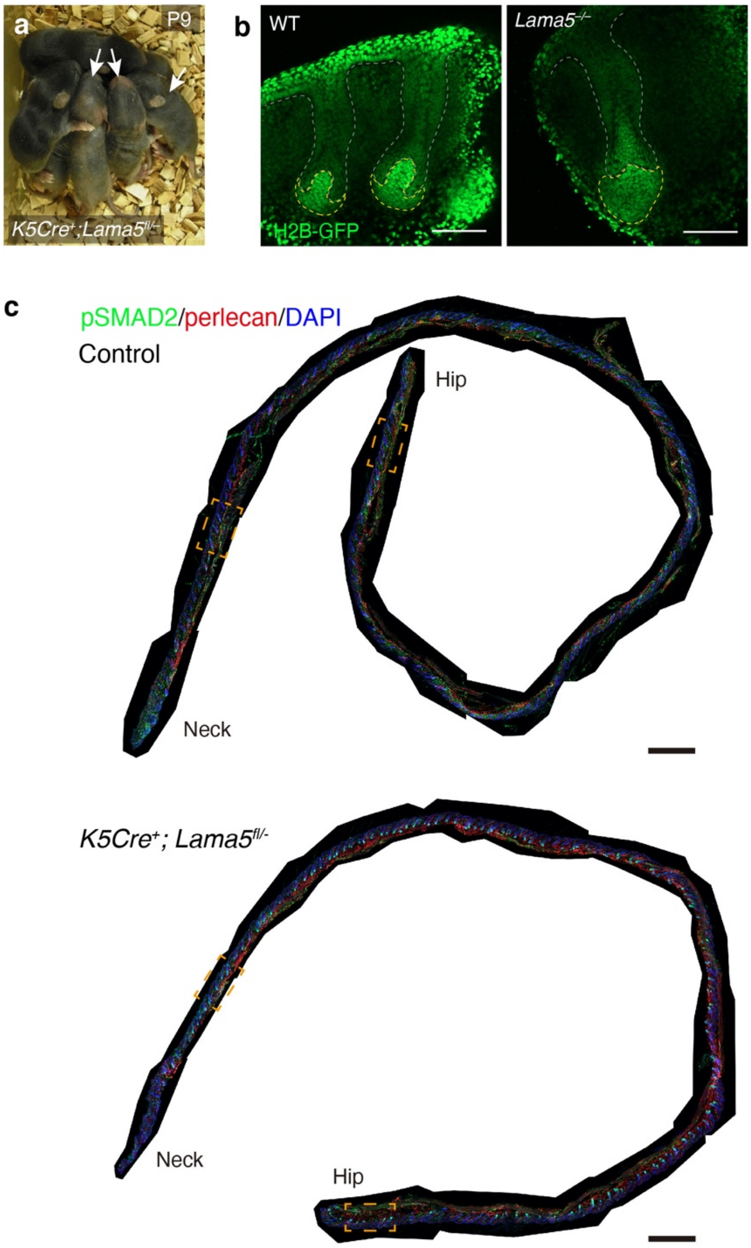

Supplementary Fig. 8 Phenotypic examination of *Lama5* mutant mice. a Gross appearance of newborn *K5Cre*$^{+}$*;Lama5*$^{fl/-}$ mice (*arrows*) with their control littermates. Hair growth is delayed. **b** Fluorescent images of developing whisker hair follicles in E14.5 R26-H2B-GFP (WT) and *R26-H2B-GFP;Lama5*$^{-/-}$ embryos. Grey dotted lines indicate the border between epithelium and dermis. Yellow dotted lines indicate the border of dermal papilla. **c** Immunohistochemical images of pSMAD2, perlecan and DAPI in dorsal skin long stripe tissues (from the neck to hip skin) from control (*K5Cre*$^{+}$*;Lama5*$^{fl/+}$) and *K5Cre*$^{+}$*;Lama5*$^{fl/-}$ mice. Orange dashed-line rectangles indicate cropped tissue regions presented in Fig. 8f. Scale bars: 100 μm (b), 1 mm (c).

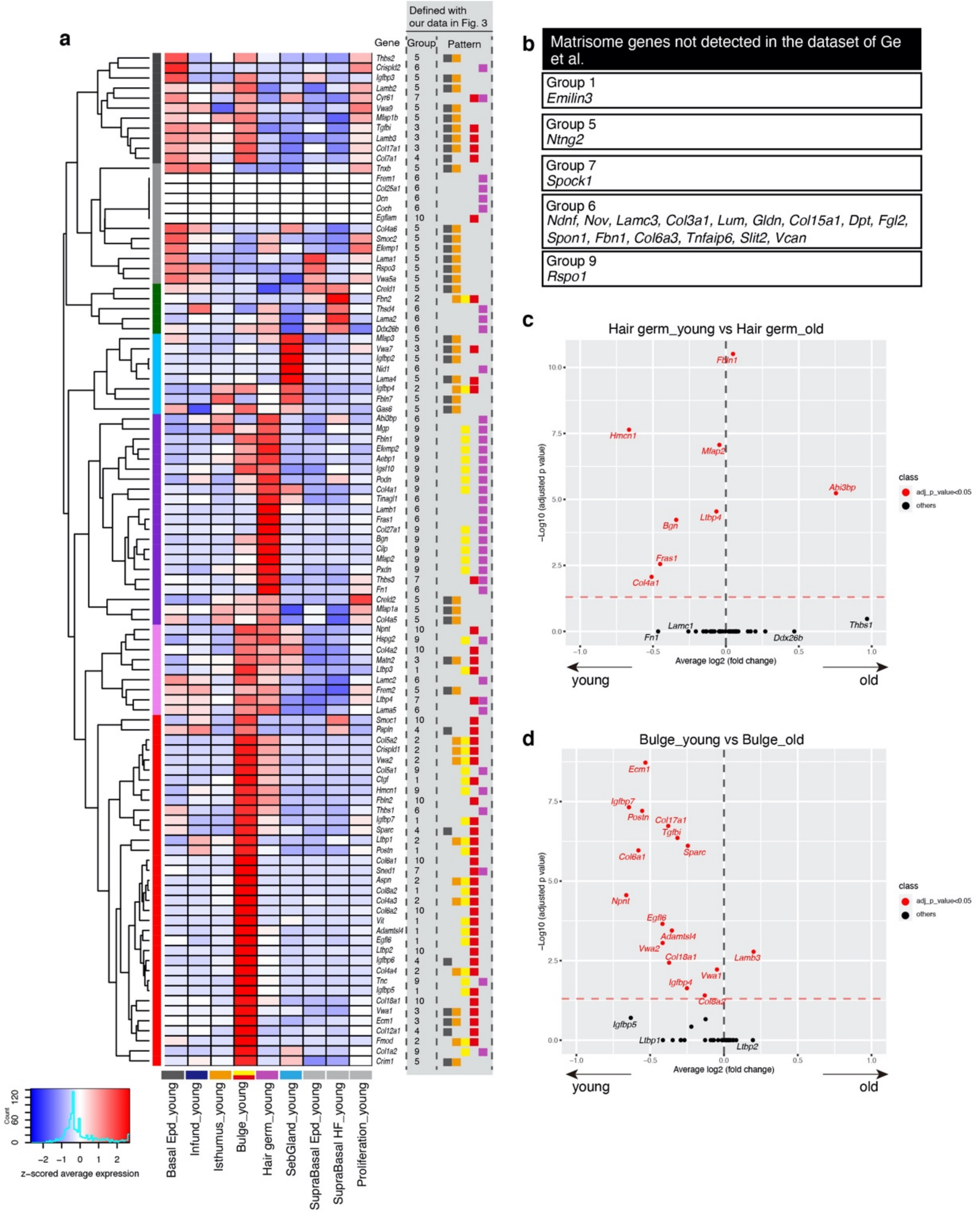

Supplementary Fig. 9 Gene expression changes of region-specific matrisome genes in aged skin. a Heatmap representing gene expression patterns of all region-specific matrisome genes, which are defined in Fig. 3 with our data, in scRNA-seq data of young skin epithelial cells of Ge et al. (2020)[2]. We analysed publicly available scRNA-seq data obtained from skin epithelial cells from 2-month-old dorsal telogen skin (Ge et al., 2020)[2] using the Seurat package. Significantly differentially expressed ECM genes were subjected to hierarchical clustering. Z-scored average expression values of each cell population are indicated according to a heat

gradient as shown at the bottom left corner. “Group” column indicates group IDs in Fig. 3. “Pattern” shows the cell populations that highly express the listed genes in our results in Fig. 3. Expression patterns of most differentially expressed ECM genes identified with the dataset of Ge et al. (2020)[2] were consistent with our results. **b** List of matrisome genes that were not detected in the analysis of the scRNA-seq dataset of Ge et al. (2020)[2], while they were detected as region-specific matrisome genes in our analysis. Each group indicates group IDs assigned in Fig. 3. **c** Volcano plot represents gene expression patterns of all hair germ (HG) ECM genes between young and aged samples. HG ECM genes (classified as epi-groups 6–9 in Fig. 3) were used for differential expression testing with MAST (Model-based Analysis of Single-cell Transcriptomics) between young and old cells in the HG[3]. The horizontal axis displays the $\log_2$ fold change values of average expression (old/young), and the vertical axis corresponds to the -$\log_{10}$ adjusted *p*-value derived from the MAST with Bonferroni correction. Vertical grey dashed line indicates fold change value = 0, and horizontal red dashed line indicates adjusted *p*-value = 0.05. Red dots indicate genes with adjusted *p*-value greater than 0.05. **d** Volcano plot represents gene expression patterns of all bulge ECM genes between young and aged samples. Bulge ECM genes (classified as epi-groups 1–4, 7 and 10 in Fig. 3) were used. Data are presented in the same way as in c.

Supplementary Table 1. List of matrisome genes analysed in this study (clustered and ordered based on their belonging gene families)

ECM Category	Gene symbol (*; proteoglycan)	Gene name	Reference
Basement membrane	*Lama1*	laminin, alpha 1	[4]
Basement membrane	*Lama2*	laminin, alpha 2	[4]
Basement membrane	*Lama3*	laminin, alpha 3	[4]
Basement membrane	*Lama4*	laminin, alpha 4	[4]
Basement membrane	*Lama5*	laminin, alpha 5	[4]
Basement membrane	*Lamb1*	laminin B1	[4]
Basement membrane	*Lamb2*	laminin, beta 2	[4]
Basement membrane	*Lamb3*	laminin, beta 3	[4]
Basement membrane	*Lamc1*	laminin, gamma 1	[4]
Basement membrane	*Lamc2*	laminin, gamma 2	[4]
Basement membrane	*Lamc3*	laminin, gamma 3	[4]
Basement membrane	*Col4a1*	collagen, type IV, alpha 1	[4]
Basement membrane	*Col4a2*	collagen, type IV, alpha 2	[4]
Basement membrane	*Col4a3*	collagen, type IV, alpha 3	[4]
Basement membrane	*Col4a4*	collagen, type IV, alpha 4	[4]
Basement membrane	*Col4a5*	collagen, type IV, alpha 5	[4]
Basement membrane	*Col4a6*	collagen, type IV, alpha 6	[4]
Basement membrane	*Col6a1*	collagen, type VI, alpha 1	[4]
Basement membrane	*Col6a2*	collagen, type VI, alpha 2	[4]
Basement membrane	*Col6a3*	collagen, type VI, alpha 3	[4]
Basement membrane	*Col6a4*	collagen, type VI, alpha 4	[4]
Basement membrane	*Col6a5*	collagen, type VI, alpha 5	[4]
Basement membrane	*Col6a6*	collagen, type VI, alpha 6	[4]
Basement membrane	*Col7a1*	collagen, type VII, alpha 1	[5]
Basement membrane	*Col8a1*	collagen, type VIII, alpha 1	[4]
Basement membrane	*Col8a2*	collagen, type VIII, alpha 2	[4]
Basement membrane	*Col15a1*	collagen, type XV, alpha 1	[4]
Basement membrane	*Col17a1*	collagen, type XVII, alpha 1	[4]
Basement membrane	*Col18a1*	collagen, type XVIII, alpha 1	[4]
Basement membrane	*Col28a1*	collagen, type XXVIII, alpha 1	[6]
Basement membrane	*Nid1*	nidogen 1	[4]
Basement membrane	*Nid2*	nidogen 2	[4]

Basement membrane	*Hspg2**	perlecan (heparan sulfate proteoglycan 2)	4
Basement membrane	*Acan**	aggrecan	7
Basement membrane	*Agrn*	agrin	4
Basement membrane	*Amelx*	amelogenin, X-linked	8
Basement membrane	*Colq*	collagen-like tail subunit (single strand of homotrimer) of asymmetric acetylcholinesterase	9
Basement membrane	*Efemp1*	epidermal growth factor-containing fibulin-like extracellular matrix protein 1	10
Basement membrane	*Egfl6*	EGF-like-domain, multiple 6	1
Basement membrane	*Egflam*	EGF-like, fibronectin type III and laminin G domains	11
Basement membrane	*Emilin3*	elastin microfibril interface 3	12
Basement membrane	*Fbln1*	fibulin 1	13
Basement membrane	*Fras1*	Fraser extracellular matrix complex subunit 1	14
Basement membrane	*Frem1*	Fras1 related extracellular matrix protein 1	14
Basement membrane	*Frem2*	Fras1 related extracellular matrix protein 2	14
Basement membrane	*Frem3*	Fras1 related extracellular matrix protein 3	15
Basement membrane	*Hmcn1*	hemicentin 1	16
Basement membrane	*Matn2*	matrilin 2	17
Basement membrane	*Mmrn2*	multimerin2	18
Basement membrane	*Npnt*	nephronectin	19
Basement membrane	*Ntn1*	netrin 1	4
Basement membrane	*Ntn3*	netrin 3	4
Basement membrane	*Ntn4*	netrin 4	4
Basement membrane	*Ntn5*	netrin 5	4
Basement membrane	*Ntng1*	netrin G1	4
Basement membrane	*Papln*	papilin, proteoglycan-like sulfated glycoprotein	18
Basement membrane	*Smoc1*	SPARC related modular calcium binding 1	18
Basement membrane	*Smoc2*	SPARC related modular calcium binding 1	18
Basement membrane	*Sparc*	secreted acidic cysteine rich glycoprotein	20
Basement membrane	*Tgfbi*	transforming growth factor, beta induced	18
Basement membrane	*Thbs2*	thrombospondin 2	21
Basement membrane	*Thbs4*	thrombospondin 4	22
Basement membrane	*Tinag*	tubulointerstitial nephritis antigen	23
Basement membrane	*Tinagl1*	tubulointerstitial nephritis antigen-like 1	24
Basement membrane	*Tnc*	tenascin C	25
Basement membrane	*Vwa1*	von Willebrand factor A domain containing 1	18
Basement membrane	*Vwa2*	von Willebrand factor A domain containing 2	26

Interstitial	*Col1a1*	collagen, type I, alpha 1	[27]
Interstitial	*Col1a2*	collagen, type I, alpha 2	[27]
Interstitial	*Col2a1*	collagen, type II, alpha 1	[27]
Interstitial	*Col3a1*	collagen, type III, alpha 1	[27]
Interstitial	*Col5a1*	collagen, type V, alpha 1	[27]
Interstitial	*Col5a2*	collagen, type V, alpha 2	[27]
Interstitial	*Col5a3*	collagen, type V, alpha 3	[27]
Interstitial	*Col9a1*	collagen, type IX, alpha 1	[27]
Interstitial	*Col9a2*	collagen, type IX, alpha 2	[27]
Interstitial	*Col9a3*	collagen, type IX, alpha 3	[27]
Interstitial	*Col10a1*	collagen, type X, alpha 1	[27]
Interstitial	*Col11a1*	collagen, type XI, alpha 1	[27]
Interstitial	*Col11a2*	collagen, type XI, alpha 2	[27]
Interstitial	*Col12a1*	collagen, type XII, alpha 1	[27]
Interstitial	*Col13a1*	collagen, type XIII, alpha 1	[27]
Interstitial	*Col14a1*	collagen, type XIV, alpha 1	[27]
Interstitial	*Col16a1*	collagen, type XVI, alpha 1	[27]
Interstitial	*Col19a1*	collagen, type XIX, alpha 1	[27]
Interstitial	*Col20a1*	collagen, type XX, alpha 1	[27]
Interstitial	*Col21a1*	collagen, type XXI, alpha 1	[27]
Interstitial	*Col22a1*	collagen, type XXII, alpha 1	[27]
Interstitial	*Col23a1*	collagen, type XXIII, alpha 1	[27]
Interstitial	*Col24a1*	collagen, type XXIV, alpha 1	[27]
Interstitial	*Col25a1*	collagen, type XXV, alpha 1	[27]
Interstitial	*Col26a1*	collagen, type XXVI, alpha 1	[27]
Interstitial	*Col27a1*	collagen, type XXVII, alpha 1	[27]
Interstitial	*Aspn**	asporin	[27]
Interstitial	*Bcan**	brevican	[27]
Interstitial	*Bgn**	biglycan	[27]
Interstitial	*Chad**	chondroadherin	[27]
Interstitial	*Chadl**	chondroadherin-like	[27]
Interstitial	*Dcn**	decorin	[27]
Interstitial	*Epyc**	epiphycan	[27]
Interstitial	*Esm1**	endothelial cell-specific molecule 1	[27]
Interstitial	*Fmod**	fibromodulin	[27]
Interstitial	*Hapln1**	hyaluronan and proteoglycan link protein 1	[27]
Interstitial	*Hapln2**	hyaluronan and proteoglycan link protein 2	[27]

Interstitial	*Hapln3**	hyaluronan and proteoglycan link protein 3	27
Interstitial	*Hapln4**	hyaluronan and proteoglycan link protein 4	27
Interstitial	*Impg1**	interphotoreceptor matrix proteoglycan 1	27
Interstitial	*Impg2**	interphotoreceptor matrix proteoglycan 2	27
Interstitial	*Kera**	keratocan	27
Interstitial	*Lum**	lumican	27
Interstitial	*Ncan**	neurocan	27
Interstitial	*Nepn**	nephrocan	27
Interstitial	*Nyx**	nyctalopin	27
Interstitial	*Ogn**	osteoglycan	27
Interstitial	*Omd**	osteomodulin	27
Interstitial	*Optc**	opticin	27
Interstitial	*Podn**	podocan	27
Interstitial	*Podnl1**	podocin-like 1	27
Interstitial	*Prelp**	proline arginine-rich and leucine-rich repeat	27
Interstitial	*Prg2**	proteoglycan 2, bone marrow	27
Interstitial	*Prg3**	proteoglycan 3	27
Interstitial	*Prg4**	proteoglycan 4 (megakaryocyte stimulating factor, articular superficial zone protein)	27
Interstitial	*Spock1**	sparc/osteonectin, cwcv and kazal-like domains proteoglycan 1	27
Interstitial	*Spock2**	sparc/osteonectin, cwcv and kazal-like domains proteoglycan 2	27
Interstitial	*Spock3**	sparc/osteonectin, cwcv and kazal-like domains proteoglycan 3	27
Interstitial	*Srgn**	serglycin	27
Interstitial	*Vcan**	versican	27
Interstitial	*5430419D17Rik (Cdcp3)*	CUB domain containing protein 3	27
Interstitial	*Abi3bp*	ABI family member 3 binding protein	27
Interstitial	*Adamtsl1*	ADAMTS-like 1	28
Interstitial	*Adamtsl2*	ADAMTS-like 2	28
Interstitial	*Adamtsl3*	ADAMTS-like 3	28
Interstitial	*Adamtsl4*	ADAMTS-like 4	28
Interstitial	*Adamtsl5*	ADAMTS-like 5	28
Interstitial	*Thsd4*	thrombospondin, type I, domain containing 4	28
Interstitial	*Adipoq*	adiponectin, C1Q and collagen domain containing	27
Interstitial	*Aebp1*	AE binding protein 1	27
Interstitial	*Ambn*	ameloblastin	27
Interstitial	*AW551984*	expressed sequence AW551984	27

Interstitial	*Bglap2*	bone gamma-carboxyglutamate protein 2	[27]
Interstitial	*Bglap3*	bone gamma-carboxyglutamate protein 3	[27]
Interstitial	*Bmper*	BMP-binding endothelial regulator	[27]
Interstitial	*Bsph1*	binder of sperm protein homolog 1	[27]
Interstitial	*Bsph2*	binder of sperm protein homolog 2	[27]
Interstitial	*Cdcp2*	CUB domain containing protein 2	[27]
Interstitial	*Cilp*	cartilage intermediate layer protein, nucleotide pyrophosphohydrolase	[27]
Interstitial	*Cilp2*	cartilage intermediate layer protein 2	[27]
Interstitial	*Coch*	cochlin	[27]
Interstitial	*Comp*	cartilage oligomeric matrix protein	[27]
Interstitial	*Creld1*	cysteine-rich with EGF-like domains 1	[27]
Interstitial	*Creld2*	cysteine-rich with EGF-like domains 2	[27]
Interstitial	*Crim1*	cysteine rich transmembrane BMP regulator 1 (chordin like)	[27]
Interstitial	*Crispld1*	cysteine-rich secretory protein LCCL domain containing 1	[27]
Interstitial	*Crispld2*	cysteine-rich secretory protein LCCL domain containing 2	[27]
Interstitial	*Ctgf (Ccn2)*	cellular communication network factor 2	[27]
Interstitial	*Cthrc1*	collagen triple helix repeat containing 1	[27]
Interstitial	*Cyr61 (Ccn1)*	cellular communication network factor 1	[27]
Interstitial	*Ddx26b (Ints6l)*	integrator complex subunit 6 like	[27]
Interstitial	*Dmbt1*	deleted in malignant brain tumors 1	[27]
Interstitial	*Dmp1*	dentin matrix protein 1	[27]
Interstitial	*Dpt*	dermatopontin	[27]
Interstitial	*Dspp*	dentin sialophosphoprotein	[27]
Interstitial	*Ecm1*	extracellular matrix protein 1	[27]
Interstitial	*Ecm2*	extracellular matrix protein 2, female organ and adipocyte specific	[27]
Interstitial	*Edil3*	EGF-like repeats and discoidin I-like domains 3	[27]
Interstitial	*Efemp2*	epidermal growth factor-containing fibulin-like extracellular matrix protein 2	[27]
Interstitial	*Egfem1*	EGF-like and EMI domain containing 1	[27]
Interstitial	*Eln*	elastin	[27]
Interstitial	*Emid1*	EMI domain containing 1	[27]
Interstitial	*Emilin1*	elastin microfibril interface 1	[27]
Interstitial	*Emilin2*	elastin microfibril interface 2	[27]
Interstitial	*Fbln2*	fibulin 2	[27]
Interstitial	*Fbln5*	fibulin 5	[27]

Interstitial	*Fbln7*	fibulin 7	[27]
Interstitial	*Fbn1*	fibrillin 1	[27]
Interstitial	*Fbn2*	fibrillin 2	[27]
Interstitial	*Fga*	fibrinogen alpha chain	[27]
Interstitial	*Fgb*	fibrinogen beta chain	[27]
Interstitial	*Fgg*	fibrinogen gamma chain	[27]
Interstitial	*Fgl1*	fibrinogen-like protein 1	[27]
Interstitial	*Fgl2*	fibrinogen-like protein 2	[27]
Interstitial	*Fn1*	fibronectin 1	[27]
Interstitial	*Fndc1*	fibronectin type III domain containing 1	[27]
Interstitial	*Fndc7*	fibronectin type III domain containing 7	[27]
Interstitial	*Fndc8*	fibronectin type III domain containing 8	[27]
Interstitial	*Gas6*	growth arrest specific 6	[27]
Interstitial	*Gldn*	gliomedin	[27]
Interstitial	*Ibsp*	integrin binding sialoprotein	[27]
Interstitial	*Igfals*	insulin-like growth factor binding protein, acid labile subunit	[27]
Interstitial	*Igfbp1*	insulin-like growth factor binding protein 1	[27]
Interstitial	*Igfbp2*	insulin-like growth factor binding protein 2	[27]
Interstitial	*Igfbp3*	insulin-like growth factor binding protein 3	[27]
Interstitial	*Igfbp4*	insulin-like growth factor binding protein 4	[27]
Interstitial	*Igfbp5*	insulin-like growth factor binding protein 5	[27]
Interstitial	*Igfbp6*	insulin-like growth factor binding protein 6	[27]
Interstitial	*Igfbp7*	insulin-like growth factor binding protein 7	[27]
Interstitial	*Igfbpl1*	insulin-like growth factor binding protein-like 1	[27]
Interstitial	*Igsf10*	immunoglobulin superfamily, member 10	[27]
Interstitial	*Kcp*	kielin/chordin-like protein	[27]
Interstitial	*Lgi1*	leucine-rich repeat LGI family, member 1	[27]
Interstitial	*Lgi2*	leucine-rich repeat LGI family, member 2	[27]
Interstitial	*Lgi3*	leucine-rich repeat LGI family, member 3	[27]
Interstitial	*Lgi4*	leucine-rich repeat LGI family, member 4	[27]
Interstitial	*Lrg1*	leucine-rich alpha-2-glycoprotein 1	[27]
Interstitial	*Ltbp1*	latent transforming growth factor beta binding protein 1	[27]
Interstitial	*Ltbp2*	latent transforming growth factor beta binding protein 1	[27]
Interstitial	*Ltbp3*	latent transforming growth factor beta binding protein 1	[27]
Interstitial	*Ltbp4*	latent transforming growth factor beta binding protein 1	[27]

Interstitial	*Matn1*	matrilin 1, cartilage matrix protein	27
Interstitial	*Matn3*	matrilin 3	27
Interstitial	*Matn4*	matrilin 4	27
Interstitial	*Mepe*	matrix extracellular phosphoglycoprotein with ASARM motif (bone)	27
Interstitial	*Mfap1a*	microfibrillar-associated protein 1A	27
Interstitial	*Mfap1b*	microfibrillar-associated protein 1B	27
Interstitial	*Mfap2*	microfibrillar-associated protein 2	27
Interstitial	*Mfap3*	microfibrillar-associated protein 3	27
Interstitial	*Mfap4*	microfibrillar-associated protein 4	27
Interstitial	*Mfap5*	microfibrillar-associated protein 5	27
Interstitial	*Mfge8*	milk fat globule EGF and factor V/VIII domain containing	27
Interstitial	*Mgp*	matrix Gla protein	27
Interstitial	*Mmrn1*	multimerin 1	27
Interstitial	*Ndnf*	neuron-derived neurotrophic factor	27
Interstitial	*Nell1*	NEL-like 1	27
Interstitial	*Nell2*	NEL-like 2	27
Interstitial	*Nov (Ccn3)*	cellular communication network factor 3	27
Interstitial	*Ntng2*	netrin G2	27
Interstitial	*Oit3*	oncoprotein induced transcript 3	27
Interstitial	*Otog*	otogelin	27
Interstitial	*Otogl*	otogelin-like	27
Interstitial	*Otol1*	otolin 1	27
Interstitial	*Pcolce*	procollagen C-endopeptidase enhancer protein	27
Interstitial	*Pcolce2*	procollagen C-endopeptidase enhancer 2	27
Interstitial	*Postn*	periostin, osteoblast specific factor	27
Interstitial	*Pxdn*	peroxidasin	27
Interstitial	*Reln*	reelin	27
Interstitial	*Rspo1*	R-spondin 1	27
Interstitial	*Rspo2*	R-spondin 2	27
Interstitial	*Rspo3*	R-spondin 3	27
Interstitial	*Rspo4*	R-spondin 4	27
Interstitial	*Sbspon*	somatomedin B and thrombospondin, type 1 domain containing	27
Interstitial	*Slamf6*	SLAM family member 6	27
Interstitial	*Slit1*	slit guidance ligand 1	27
Interstitial	*Slit2*	slit guidance ligand 2	27

Interstitial	*Slit3*	slit guidance ligand 3	27
Interstitial	*Sned1*	sushi, nidogen and EGF-like domains 1	27
Interstitial	*Sparcl1*	SPARC-like 1	27
Interstitial	*Spon1*	spondin 1, (f-spondin) extracellular matrix protein	27
Interstitial	*Spon2*	spondin 2, (f-spondin) extracellular matrix protein	27
Interstitial	*Spp1*	secreted phosphoprotein 1	27
Interstitial	*Srpx*	sushi-repeated-containing protein	27
Interstitial	*Srpx2*	sushi-repeated-containing protein, X-linked 2	27
Interstitial	*Sspo*	SCO-spondin	27
Interstitial	*Svep1*	sushi, von Willebrand factor type A, EGF and pentraxin domain containing 1	27
Interstitial	*Tecta*	tectorin alpha	27
Interstitial	*Tectb*	tectorin beta	27
Interstitial	*Thbs1*	thrombospondin 1	27
Interstitial	*Thbs3*	thrombospondin 3	27
Interstitial	*Tnfaip6*	tumor necrosis factor alpha induced protein 6	27
Interstitial	*Tnn*	tenascin N	27
Interstitial	*Tnr*	tenascin R	27
Interstitial	*Tnxb*	tenascin XB	27
Interstitial	*Tsku*	tsukushi, small leucine rich proteoglycan	27
Interstitial	*Vit*	vitrin	27
Interstitial	*Vtn*	vitronectin	27
Interstitial	*Vwa3a*	von Willebrand factor A domain containing 3A	27
Interstitial	*Vwa5a*	von Willebrand factor A domain containing 5A	27
Interstitial	*Vwa5b1*	von Willebrand factor A domain containing 5B1	27
Interstitial	*Vwa5b2*	von Willebrand factor A domain containing 5B2	27
Interstitial	*Vwa7*	von Willebrand factor A domain containing 7	27
Interstitial	*Vwa9 (Ints14)*	integrator complex subunit 14	27
Interstitial	*Vwc2*	von Willebrand factor C domain containing 2	27
Interstitial	*Vwce*	von Willebrand factor C and EGF domains	27
Interstitial	*Vwde*	von Willebrand factor D and EGF domains	27
Interstitial	*Vwf*	Von Willebrand factor	27
Interstitial	*Wisp1 (Ccn4)*	cellular communication network factor 4	27
Interstitial	*Wisp2 (Ccn5)*	cellular communication network factor 5	27
Interstitial	*Wisp3 (Ccn6)*	cellular communication network factor 6	27

Interstitial	*Zp1*	zona pellucida glycoprotein 1	[27]
Interstitial	*Zp2*	zona pellucida glycoprotein 2	[27]
Interstitial	*Zp3*	zona pellucida glycoprotein 3	[27]
Interstitial	*Zp3r*	zona pellucida 3 receptor	[27]
Interstitial	*Zpld1*	zona pellucida like domain containing 1	[27]

Supplementary Table 2. Primer sequences used for qRT-PCR

Target genes	Primer sequence (forward)	Primer sequence (reverse)
Gapdh	TGCCAGCCTCGTCCCGTAG	CGGCCTTGACTGTGCCGTTG
Itga6	CGGGCACTCAGGTTCGAGTGA	AGCCTTGTGATAGGTGGCATCGT
Lrig1	TGTGTGTCTGAGAGACCCGAGC	CAGAGCCACTGTGTGCTGTTGT
Lgr6	GGCTCCGAATCCTGGAGCTGT	CTCAGGGTGGATGGCACGGA
Gli1	ATCTCCGGGCGGTTCCTACG	TGACTTCAGCTGGCAGGTTGC
Bdnf	GCGGCGCCCATGAAAGAAGT	GGCCGAACCTTCTGGTCCTCA
Cd34	TGGCGCTGGGTAGCTCTCTG	TAGTCTCTGAGATGGCTGGTGTGG
Cdh3	TGGGGAAAGTAGCCTTGGCTGG	CAGCCTCTGAGGGAAGGGACC
Lef1	GCCGGGATGCCCCAACTTTC	GACCACCTCATGCCCGTTGC
Pdgfra	TGCGGGTTTTGAGCCCATTACT	CCGGCCCTGTGAGGAGACAG
Itga8	TCTTGTGCAGTGGGTCGCCT	CCGACGTCTTAACCGCTGTGC
eGFP	TGGTGCCCATCCTGGTCGAC	GAAGCACTGCACGCCGTAGG

Supplementary Table 3. RNA-seq read and mapping statistics

	Raw Read Count	***Trimmed Read Count***	***rDNA Read Count***	***Multi-Mapped Read Count***	***Uniquely mapped Read Count***
Mouse_Basal_ Cells_Rep1	11,757,494	11,278,061	148,700	170,077	10,456,332
Mouse_Basal_ Cells_Rep2	11,245,045	10,668,752	73,729	134,777	10,051,376
Mouse_Basal_ Cells_Rep3	11,313,239	10,747,103	108,596	146,602	10,089,518
Mouse_Bulge_ StemCells_Rep1	11,735,034	11,181,377	64,614	183,557	10,520,864
Mouse_Bulge_ StemCells_Rep2	11,917,049	11,515,851	54,154	175,176	10,889,932
Mouse_Bulge_ StemCells_Rep3	13,417,100	12,895,113	88,655	218,495	12,090,872
Mouse_Cdh3+_ Cells_Rep1	10,875,593	10,409,342	68,193	177,407	9,574,901
Mouse_Cdh3+_ Cells_Rep2	11,565,616	10,993,093	65,001	185,981	10,079,620
Mouse_Cdh3+_ Cells_Rep3	11,405,265	10,746,768	76,143	206,384	9,790,096
Mouse_Cdh3+_ Cells_Rep4	11,759,881	11,186,543	85,902	191,154	10,274,371
Mouse_Gli1+_ Cells_Rep1	12,063,488	11,478,537	57,630	178,646	10,751,521
Mouse_Gli1+_ Cells_Rep2	19,762,234	18,804,713	178,685	303,848	17,446,776
Mouse_Gli1+_ Cells_Rep3	19,543,117	18,568,695	122,017	287,214	17,378,904
Mouse_Lgr6+_ Cells_Rep1	20,044,455	19,058,807	121,507	307,804	17,798,594
Mouse_Lgr6+_ Cells_Rep2	19,756,354	18,736,071	197,458	298,652	17,386,391
Mouse_Lgr6+_ Cells_Rep3	19,639,299	18,680,969	217,502	298,146	17,210,635
Mouse_Lef1+_ Cells_Rep1	13,353,072	12,447,662	90,361	163,390	11,573,835
Mouse_Lef1+_ Cells_Rep2	11,534,943	10,876,371	69,732	150,330	10,020,573
Mouse_Lef1+_ Cells_Rep3	11,479359	10,679,409	55,674	123,456	10,001,747
Mouse_Lef1+_ Cells_Rep4	10,991,615	10,405,782	42,283	117,783	9,796,296
Mouse_Pdgfa+_ Cells_Rep1	21,043,519	20,002,460	197,994	308,101	18,559,766
Mouse_Pdgfa+_ Cells_Rep2	20,727,828	19,678415	211,148	324,166	18,190,226
Mouse_Pdgfa+_ Cells_Rep3	20,638,004	19,549,144	214,785	339,207	17,998,448

References

1. Cheng, C. C. *et al.* Hair follicle epidermal stem cells define a niche for tactile sensation. *Elife* **7**, e38883 (2018).

2. Ge, Y. *et al.* The aging skin microenvironment dictates stem cell behavior. *Proc. Natl. Acad. Sci. USA* **117**, 5339–5350 (2020).

3. Finak, G. *et al.* Mast: A flexible statistical framework for assessing transcriptional changes and characterizing heterogeneity in single-cell rna sequencing data. *Genome Biol.* **16**, 278 (2015).

4. Yurchenco, P. D. Basement membranes: Cell scaffoldings and signaling platforms. *Cold Spring Harb. Perspect. Biol.* **3**, a004911 (2011).

5. Heinonen, S. *et al.* Targeted inactivation of the type vii collagen gene (col7a1) in mice results in severe blistering phenotype: A model for recessive dystrophic epidermolysis bullosa. *J. Cell Sci.* **112 (Pt 21)**, 3641–3648 (1999).

6. Veit, G. *et al.* Collagen xxviii, a novel von willebrand factor a domain-containing protein with many imperfections in the collagenous domain. *J. Biol. Chem.* **281**, 3494–3504 (2006).

7. Zanin, M. K. *et al.* Distinct spatial and temporal distributions of aggrecan and versican in the embryonic chick heart. *Anat. Rec.* **256**, 366–380 (1999).

8. Fukumoto, S., Yamada, A., Nonaka, K. & Yamada, Y. Essential roles of ameloblastin in maintaining ameloblast differentiation and enamel formation. *Cells Tissues Organs* **181**, 189–195 (2005).

9. Maselli, R. A., Arredondo, J., Ferns, M. J. & Wollmann, R. L. Synaptic basal lamina-associated congenital myasthenic syndromes. *Ann. N Y Acad. Sci.* **1275**, 36–48 (2012).

10. Vukovic, J. *et al.* Lack of fibulin-3 alters regenerative tissue responses in the primary olfactory pathway. *Matrix Biol.* **28**, 406–415 (2009).

11. Omori, Y. *et al.* Presynaptic dystroglycan-pikachurin complex regulates the proper synaptic connection between retinal photoreceptor and bipolar cells. *J. Neurosci.* **32**, 6126–6137 (2012).

12. Schiavinato, A. *et al.* Emilin-3, peculiar member of elastin microfibril interface-located protein (emilin) family, has distinct expression pattern, forms oligomeric assemblies, and serves as transforming growth factor beta (tgf-beta) antagonist. *J. Biol. Chem.* **287**, 11498–11515 (2012).

13. Kluge, M., Mann, K., Dziadek, M. & Timpl, R. Characterization of a novel calcium-binding 90-kda glycoprotein (bm-90) shared by basement membranes and serum. *Eur. J. Biochem.* **193**, 651–659 (1990).

14. Kiyozumi, D., Sugimoto, N. & Sekiguchi, K. Breakdown of the reciprocal stabilization of qbrick/frem1, fras1, and frem2 at the basement membrane provokes fraser syndrome-like defects. *Proc. Natl. Acad. Sci. USA* **103**, 11981–11986 (2006).

15. Kiyozumi, D., Sugimoto, N., Nakano, I. & Sekiguchi, K. Frem3, a member of the 12 cspg repeats-containing extracellular matrix protein family, is a basement membrane protein with tissue distribution patterns distinct from those of fras1, frem2, and qbrick/frem1. *Matrix Biol.* **26**, 456–462 (2007).

16. Morrissey, M. A. *et al.* B-link: A hemicentin, plakin, and integrin-dependent adhesion system that links tissues by connecting adjacent basement membranes. *Dev. Cell* **31**, 319–331 (2014).

17. Piecha, D. *et al.* Matrilin-2 interacts with itself and with other extracellular matrix proteins. *Biochem. J.* **367**, 715–721 (2002).

18. Manabe, R. *et al.* Transcriptome-based systematic identification of extracellular matrix proteins. *Proc. Natl. Acad. Sci. USA* **105**, 12849–12854 (2008).

19. Fujiwara, H. *et al.* The basement membrane of hair follicle stem cells is a muscle cell niche. *Cell* **144**, 577–589 (2011).

20. Mayer, U., Aumailley, M., Mann, K., Timpl, R. & Engel, J. Calcium-dependent binding of basement membrane protein bm-40 (osteonectin, sparc) to basement membrane collagen type iv. *Eur. J. Biochem.* **198**, 141–150 (1991).

21. Tian, W., Sawyer, A., Kocaoglu, F. B. & Kyriakides, T. R. Astrocyte-derived thrombospondin-2 is critical for the repair of the blood-brain barrier. *Am. J. Pathol.* **179**, 860–868 (2011).

22. Si, Z. *et al.* Distribution of thrombospondin-4 in the bovine eye. *Curr. Eye Res.* **27**, 165–173 (2003).

23. Xie, P. *et al.* Role of extracellular matrix renal tubulo-interstitial nephritis antigen (tinag) in cell survival utilizing integrin (alpha)vbeta3/focal adhesion kinase (fak)/phosphatidylinositol 3-kinase (pi3k)/protein kinase b-serine/threonine kinase (akt) signaling pathway. *J. Biol. Chem.* **286**, 34131–34146 (2011).

24. Igarashi, T. *et al.* Tubulointerstitial nephritis antigen-like 1 is expressed in extraembryonic tissues and interacts with laminin 1 in the reichert membrane at postimplantation in the mouse. *Biol. Reprod.* **81**, 948–955 (2009).

25. Koch, M. *et al.* Epithelial synthesis of tenascin at tips of growing bronchi and graded accumulation in basement membrane and mesenchyme. *Exp. Cell Res.* **194**, 297–300 (1991).

26. Sengle, G., Kobbe, B., Morgelin, M., Paulsson, M. & Wagener, R. Identification and characterization of amaco, a new member of the von willebrand factor a-like domain protein superfamily with a regulated expression in the kidney. *J. Biol. Chem.* **278**, 50240–50249 (2003).

27. Naba, A. *et al.* The matrisome: In silico definition and in vivo characterization by proteomics of normal and tumor extracellular matrices. *Mol. Cell. Proteomics* **11**, M111 014647 (2012).

28. Tsutsui, K. *et al.* Adamtsl-6 is a novel extracellular matrix protein that binds to fibrillin-1 and promotes fibrillin-1 fibril formation. *J. Biol. Chem.* **285**, 4870–4882 (2010).
