# Supplementary material for: Mapping the molecular and structural specialization of the skin basement membrane for inter-tissue interactions
Source: Nat Commun. 2021 May 10;12:2577. doi: 10.1038/s41467-021-22881-y (PMC8110968; doi:10.1038/s41467-021-22881-y)
Supplement: Supplementary file 3 — Description of Additional Supplementary Files [file 41467_2021_22881_MOESM3_ESM.pdf]

Description of Additional Supplementary Files

File Name: Supplementary Data 1
Description: File Name: Supplementary Data 1
Description: Antibodies used in this study, their specific dilutions and tissue fixation methods

File Name: Supplementary Data 2
Description: Consistencies and discrepancies in tissue localization between transcripts and proteins involved in ECMs.

File Name: Supplementary Movie 1
Description: High-resolution three-dimensional reconstruction of the hook basement membrane
