# Supplementary material for: Mapping the molecular and structural specialization of the skin basement membrane for inter-tissue interactions
Source: Nat Commun. 2021 May 10;12:2577. doi: 10.1038/s41467-021-22881-y (PMC8110968; doi:10.1038/s41467-021-22881-y)
Supplement: Supplementary file 7 — Reporting Summary [file 41467_2021_22881_MOESM7_ESM.pdf]

nature research

Corresponding author(s): Hironobu Fujiwara

Last updated by author(s): Mar 19, 2021

Reporting Summary

Nature Research wishes to improve the reproducibility of the work that we publish. This form provides structure for consistency and transparency in reporting. For further information on Nature Research policies, seeAuthors & Referees and theEditorial Policy Checklist .

Statistics

For all statistical analyses, confirm that the following items are present in the figure legend, table legend, main text, or Methods section.

n/a	Confirmed	
☐	☒	The exact sample size (*n*) for each experimental group/condition, given as a discrete number and unit of measurement
☐	☒	A statement on whether measurements were taken from distinct samples or whether the same sample was measured repeatedly
☐	☒	The statistical test(s) used AND whether they are one- or two-sided<br>*Only common tests should be described solely by name; describe more complex techniques in the Methods section.*
☒	☐	A description of all covariates tested
☒	☐	A description of any assumptions or corrections, such as tests of normality and adjustment for multiple comparisons
☐	☒	A full description of the statistical parameters including central tendency (e.g. means) or other basic estimates (e.g. regression coefficient) AND variation (e.g. standard deviation) or associated estimates of uncertainty (e.g. confidence intervals)
☐	☒	For null hypothesis testing, the test statistic (e.g. *F*, *t*, *r*) with confidence intervals, effect sizes, degrees of freedom and *P* value noted<br>*Give P values as exact values whenever suitable.*
☒	☐	For Bayesian analysis, information on the choice of priors and Markov chain Monte Carlo settings
☐	☒	For hierarchical and complex designs, identification of the appropriate level for tests and full reporting of outcomes
☒	☐	Estimates of effect sizes (e.g. Cohen's *d*, Pearson's *r*), indicating how they were calculated

Our web collection on statistics for biologists contains articles on many of the points above.

Software and code

Policy information about availability of computer code

Data collection	This does not apply to this study.
Data analysis	All the flow cytometry data were collected by FACSAria II and analyzed by FACSDiva (v7 and v8.0) and FlowJo (v9.9.3); qPCR data were analyzed by Excel 2016; RNA-seq data were analyzed using HiSeq (v2.2.58), RTA (v1.18.64), bcl2fastq (v1.8.4), FastQC (v0.11.3), Trim Galore (v0.6.5), HISAT2 (v2.1.0), StringTie (2.0.4); Gene expression analysis were performed with DEseq2 (1.28.1), Seurat (v2.3.4) and other Bioconductor packages in R (v4.0.2); GO data were analyzed by Panther (14.1); Charts of hierarchical clustering, expression correlation and principal component analysis were plotted with bioconductor R using heatmap.2 function in ggplot2 (v3.3.2) and corrplot (v0.84); Gene set enrichment analysis was performed with GSEA (v4.1.0); image quantitative data were analyzed by using Imaris (v9.3) and Fiji ImageJ (v2.0). R codes used in this study are available in GitHub: https://github.com/FujiwaraLab/Tsutsui_etal with DOI: 10.5281/zenodo.4620935 [https://doi.org/10.5281/zenodo.4620935].

For manuscripts utilizing custom algorithms or software that are central to the research but not yet described in published literature, software must be made available to editors/reviewers. We strongly encourage code deposition in a community repository (e.g. GitHub). See the Nature Research guidelines for submitting code & software for further information.

Data

Policy information about availability of data

All manuscripts must include a data availability statement. This statement should provide the following information, where applicable:

- Accession codes, unique identifiers, or web links for publicly available datasets
- A list of figures that have associated raw data
- A description of any restrictions on data availability

All data that support the findings of this study are available within the paper and its supplementary information files or are available from the corresponding author upon reasonable request. The RNA-seq data used and reported in this study are available in BioProject PRJNA342736 [https://www.ncbi.nlm.nih.gov/bioproject/342736] and PRJDB9477 [https://www.ncbi.nlm.nih.gov/bioproject/?term=PRJDB9477]. Source data are provided with this paper.

Field-specific reporting

Please select the one below that is the best fit for your research. If you are not sure, read the appropriate sections before making your selection.

- [x] Life sciences
- [ ] Behavioural & social sciences
- [ ] Ecological, evolutionary & environmental sciences

For a reference copy of the document with all sections, see nature.com/documents/nr-reporting-summary-flat.pdf

Life sciences study design

All studies must disclose on these points even when the disclosure is negative.

Sample size	No statistical methods were used to predetermine sample sizes. Sample sizes were determined based on previous publications on similar experiments (e.g. Cheng et al., eLife 7, e38883 (2018))
Data exclusions	No data were excluded for this study.
Replication	For quantitative analysis, we have three or more biological replicates for each experiment, except for Figure 9c and Supplemental Figure 1l, and reproduced with similar results. We show representative micrographs that came from at least two biological replicates.
Randomization	Samples were allocated to groups of a given genotype, detected by standard genetic/genomic approaches. Otherwise samples were randomly selected within these groups.
Blinding	In most of the experiments, no blinding method was possible as only one experimenter was performing the analysis.

Reporting for specific materials, systems and methods

We require information from authors about some types of materials, experimental systems and methods used in many studies. Here, indicate whether each material, system or method listed is relevant to your study. If you are not sure if a list item applies to your research, read the appropriate section before selecting a response.

Materials & experimental systems

n/a	Involved in the study	
☐	☒	Antibodies
☒	☐	Eukaryotic cell lines
☒	☐	Palaeontology
☐	☒	Animals and other organisms
☒	☐	Human research participants
☒	☐	Clinical data

Methods

n/a	Involved in the study	
☒	☐	ChIP-seq
☐	☒	Flow cytometry
☒	☐	MRI-based neuroimaging

Antibodies

Antibodies used

We have described all antibodies used in this study in the manuscript (Supplementary Data 1) and here in the Reporting Summary, including their suppliers, catalogue numbers, clone numbers and dilutions.

Antibody list:

-FACS-

CD45-PE-Cy7, Rat, eBioscience, 30-F11, FACS (1:100); TER-119-PE-Cy7, Rat, eBioscience, TER119, FACS (1:100); CD31-PE-Cy7, Rat, eBioscience, 390, FACS (1:100); Sca-1-PerCP-Cy5.5, Rat, eBioscience, D7, FACS (1:100); CD34-eFluor660, Rat, eBioscience, RAM34, FACS (1:100); CD49f-PE, Rat, eBioscience, GoH3, FACS (1:100)

-ECM-related-

ABI3BP, Rabbit, Sekiguchi lab., serum, IHC (1:8); adamtsl-6, Rabbit, Sekiguchi lab., serum, IHC (1:500); agrin, Rabbit, Santa Cruz, sc-25528, IHC (5 μg/ml); asporin, Rabbit, Atlas, Antibodies, HPA024230, IHC (10 μg/ml); asporin, Rabbit, abcam, ab58741, IHC (25 μg/ml); biglycan, Goat, R&D, AF2667, IHC (10 μg/ml); CD34, Rat, Hycult Biotech, HM1015 (MEC14.7), IHC (2 μg/ml); CD34, Rabbit, abcam, ab81289, IHC (10.3 μg/ml); collagen III, Rabbit, abcam, ab7778, IHC (50 μg/ml); COL4A1, Rat, Shigei Med. Res. Inst., H11, IHC (1:20); COL4A2, Rat, Shigei Med. Res. Inst., H22, IHC (1:20); COL4A3, Rat, Shigei Med. Res. Inst., H31, IHC (1:10); COL4A4, Rat, Shigei Med. Res. Inst., RH42, IHC (1:10); COL4A5, Rat, Shigei Med. Res. Inst., H53, IHC (1:10); COL4A6, Rat, Shigei Med. Res. Inst., B66, IHC (1:10); collagen VI, Rabbit, abcam, ab6588, IHC (50 μg/ml); COL6A1, Rabbit, Sigma, HPA029401, IHC (2.5 μg/ml); COL6A2, Rabbit, Santa Cruz, sc-8360, IHC (1 μg/ml); COL6A3, Rabbit, Abnova, PAB17517, IHC (10 μg/ml); COL6A3, Rabbit, Sigma, HPA010080, IHC (1.25 μg/ml); COL6A6, Rabbit, Novus, NBP2-14546, IHC (30 μg/ml); COL6A6, Rabbit, Sigma, HPA045239, IHC (1 μg/ml); COL7A1, Rabbit, LSBio, LS-B10205, IHC (20 μg/ml); COL7A1, Rabbit, Merck, 234192, IHC (1:40); COL8A2, Rabbit, USBiological, 34099, IHC (2.3 or 11.4 μg/ml); COL12A1, Rabbit, St John's, STJ92375, IHC (12.5 μg/ml); COL13A1, Rabbit, Atlas Antibodies, HPA050392, IHC (2.5 μg/ml); COL13A1, Sheep, R&D, AF4627, IHC (5.6 μg/ml); COL17A1, Mouse, Hirako Lab, R311, IHC (no dilution); COL18A1, Goat, R&D, AF1098, IHC (4 μg/ml); COL23A1, Rabbit, Novus, NBP2-38111, IHC (2.5 μg/ml); COL27A1, Rabbit, abcam, ab179753, IHC (25 μg/ml); COL27A1, Rabbit, Thermo Fisher, PA5-54535, IHC (2.5 μg/ml); CRIM1, Rabbit, Atlas

Antibodies, HPA000556, IHC (5 or 2 μg/ml); CRIM1, Goat, R&D, AF1917, IHC (2 μg/ml); CRIM1, Rabbit, GeneTex, GTX51530, IHC (10 μg/ml); CRIM1, Rabbit, Fujiwara Lab. (this paper), serum, IHC (1:400); CRISPLD1, Rabbit, abcam, ab123039, IHC (25 μg/ml); CTGF, Rabbit, abcam, ab6992, IHC (25 μg/ml); CTGF, Goat R&D, AF660, IHC (12.6 μg/ml); decorin, Goat, R&D, AF1060, IHC (2 μg/ml); dermatopontin, Rabbit, LSBio, LS-C167524, IHC (1:20); dermatopontin, Rabbit, Cloud-Clone, PAC432Hu01, IHC (20 μg/ml); ECM1, Rabbit, Proteintech, 11521-AP, IHC (9.3 or 1.9 μg/ml); ECM1, Goat, R&D, AF4428, IHC (0.4 or 4 μg/ml); ECM1, Sheep, R&D, AF3937, IHC (2 μg/ml); EDIL3, Rabbit, abcam, ab198003, IHC (1:100); EDIL3, Rabbit, Proteintech, 12580-1-AP, IHC (6.7 μg/ml); EGFL6, Rabbit, Fujiwara Lab., CUK-1203-022, IHC (1 μg/ml); fibronectin, Rabbit, abcam, ab23750, IHC (4 μg/ml); fibulin-2, Rabbit, Atlas Antibodies, HPA001934, IHC (10 μg/ml); FRAS1, Rabbit, Santa Cruz, sc-98444, IHC (10 or 2 μg/ml); IGFBP5, Goat, R&D, AF578, IHC (1 μg/ml); IGFBP6, Rabbit, Santa Cruz, sc-13094, IHC (5 μg/ml); IGFBP7, Goat, abcam, ab129302, IHC (25 μg/ml); IGFBP7, Rabbit, Thermo Fisher, PA5-76685, IHC (50 μg/ml); integrin α3, Goat, R&D, AF2787, IHC (2 μg/ml); integrin α5, Rabbit, abcam, ab221606, IHC (2.1 μg/ml); integrin α6, Rat, Biolegend, GoH3, IHC (10 μg/ml); integrin α8, Goat, R&D, AF4076, IHC (0.8 μg/ml); integrin α9, Goat, R&D, AF3827, IHC (2 μg/ml); integrin αv, Goat, R&D, AF1219, IHC (10 μg/ml); integrin β1, Hamster, Biolegend, HMb1-1, IHC (10 μg/ml); integrin β4, Rabbit, Sigma, HPA036348, IHC (2 μg/ml); integrin β8, Rabbit, Santa Cruz, sc-25714, IHC (5 μg/ml); laminin-332, Rabbit, abcam, ab14509, IHC (5 μg/ml); laminin α2, Rat, abcam, 4H8-2, IHC (20.8 μg/ml); laminin α3, Rabbit, LSBio, LS-C670558, IHC (8.1 μg/ml); laminin α4, Goat, R&D, AF3837, IHC (5 μg/ml); laminin α5, Rabbit, Fujiwara Lab. (this paper), CUK-1185-006, IHC (0.09 μg/ml); laminin β1, Rat, GeneTex, LT3, IHC (0.7 μg/ml); laminin β2, Mouse, abcam, ab210956, IHC (1:10); laminin β2, Rat, Sekiguchi lab., B24-N8-D6, IHC (3.7 μg/ml); laminin γ1, Rat, Merck, MAB1914P, IHC (1 μg/ml); laminin γ3, Rabbit, Santa Cruz, sc-25719, IHC (2 or 4 μg/ml); laminin γ3, Rat, Sekiguchi lab., C38-N4-F4, IHC (3.3 μg/ml); LTBP2, Rabbit, Santa Cruz, sc-48759, IHC (5 μg/ml); LTBP3, Rabbit, Merck, ABT316, IHC (12.5 μg/ml); LTBP3, Mouse, Santa Cruz, sc-390913, AF488, IHC (10 μg/ml); matrilin-1, Rabbit, Atlas Antibodies, HPA028580, IHC (2.5 μg/ml); matrilin-2, Goat, R&D, AF3044, IHC (25.1 μg/ml); NDNF, Rabbit, Abgent, AP5812a, IHC (25 μg/ml); NDNF, Goat, Santa Cruz, sc-242196, IHC (5 μg/ml); NDNF, Rabbit, LSBio, LS-C319946, IHC (6.4 μg/ml); nephronectin, Rabbit, Fujiwara lab., CUK-1192-006, IHC (2.25 μg/ml); netrin-4, Goat, R&D, AF1132, IHC (5 μg/ml); nidogen-1, Rat, Merck, MAB1886, IHC (4 μg/ml); nidogen-2, Goat, R&D, AF3385, IHC (0.8 μg/ml); osteopontin, Rabbit, abcam, ab8448, IHC (1:500); papilin, Rabbit, Sekiguchi lab., serum, IHC (1:40); periostin, Rabbit, abcam, ab14041, IHC (12.5 μg/ml); perlecan, Rat, Merck, MAB1948P, IHC (1:500); peroxidasin, Goat, Santa Cruz, sc-168598, IHC (0.2 μg/ml); plectin, Guinea pig, Progen, GP21, IHC (1:1,000); R-spondin-1, Rabbit, Santa Cruz, sc-49092-R, IHC (10 μg/ml); R-spondin-1, Rabbit, Atlas Antibodies, HPA046154, IHC (20 μg/ml); R-spondin-1, Goat, R&D, AF3474, IHC (1 or 2 μg/ml); R-spondin-2, Rat, R&D, 879712R, IHC (2.5 μg/ml); R-spondin-3, Rabbit, Proteintech, 17193-1-AP, IHC (9.3 or 3.7 μg/ml); serglycin, Mouse, Santa Cruz, C-11, IHC (5 μg/ml); serglycin, Mouse, Abnova, H00005552-M03, IHC (12 μg/ml); SMOC1, Goat, R&D, AF5550, IHC (25 μg/ml); SNED1, Rabbit, Atlas Antibodies, HPA036415, IHC (2.5 μg/ml); SPARC, Rabbit, abcam, ab14174, IHC (1:200); SPARC, Rat, R&D, MAB942-SP, IHC (25 μg/ml); SPARC, Goat, R&D, AF941, IHC (5 μg/ml); SPOCK1, Rabbit, Proteintech, 12512-1-AP, IHC (11 μg/ml); spondin-1, Rabbit, abcam, ab40797, IHC (2 μg/ml); spondin-1, Goat, R&D, AF3135, IHC (0.4 μg/ml); tenascin-C, Rat, Merck, AB19013, IHC (1.25 μg/ml); tenascin-N, Rabbit, abcam, ab121887, IHC (5 μg/ml); TGFBI, Rabbit, Sekiguchi lab., serum, IHC (1:200); THBS1, Mouse, Santa Cruz, sc-59887, IHC (2 μg/ml); TNFAIP6, Rabbit, Sigma, HPA050884, IHC (10 μg/ml); TNFAIP6, Rabbit, Thermo Fisher, PA5-62259, IHC (2 μg/ml); vitrin, Rabbit, Novus, NBP2-49453, IHC (2.5 μg/ml); VWA1, Rabbit, Sekiguchi lab., serum, IHC (1:100)

-Secondary-

Goat IgG Alexa 555, Donkey, Life Technologies, A21432, IHC (1:500); Guinea pig IgG Alexa 488, Goat, Life Technologies, A11073, IHC (1:500); Hamster IgG Alexa 546, Goat, Life Technologies, A21111, IHC (1:500); Mouse IgG2a Alexa 488, Goat, Life Technologies, A21131, IHC (1:500); Mouse IgM Alexa 488, Goat, Life Technologies, A21042, IHC (1:500); Rabbit IgG Alexa 488, Goat, Life Technologies, A11034, IHC (1:500); Rabbit IgG Alexa 555, Goat, Life Technologies, A21429, IHC (1:500); Rat IgG Alexa 488, Donkey, Life Technologies, A21208, IHC (1:500); Rat IgG Alexa 488, Goat, Life Technologies, A11006, IHC (1:500); Rat IgG Alexa 555, Goat, Life Technologies, A21434, IHC (1:500); Sheep IgG Alexa 647, Donkey, Life Technologies, A21448, IHC (1:500)

Validation

For in house antibodies against laminin α5, we have validated the antibody specificity with tissue samples from knockout mice of the gene. For in house antibodies against CRIM1, we have confirmed the same staining pattern as that have been reported previously by other research groups using mouse embryonic skin. For commercial antibodies, we have selected antibodies that have been broadly used in the field and have enough publication records. The validation of these commercial antibodies can be found on the manufacture's website. We have also carefully validated the antibody specificity with their subcellular and tissue localization patterns in our analyses. For ECM antibodies, in addition to the criteria described above, we have also validated the antibody specificity with their mRNA expression patterns in the skin.

Animals and other organisms

Policy information about studies involving animals; ARRIVE guidelines recommended for reporting animal research

Laboratory animals

We have obtained following animals from indicated institutions. Age and sex of the animals used for each experiments are described in the appropriate positions in the main text of this manuscript. In general, we used female 8-week-old mice for experiments, unless otherwise specified. Mouse: C57BL/6N (Japan SLC Inc.), Mouse: Lgr6-GFP-ires-CreERT2 (Jackson Laboratories; 016934), Mouse: Gli1-eGFP (MMRRC; STOCK Tg(Gli1-EGFP)DM197Gsat/Mmucd), Mouse: Cdh3-eGFP (MMRRC; STOCK Tg(Cdh3-EGFP)BK102Gsat/Mmnc), Mouse: Lef1-eGFP (MMRRC; STOCK Tg(Lef1-EGFP)IN75Gsat/Mmucd), Mouse: Pdgfra-H2B-eGFP (Jackson Laboratories; 007669), Mouse: keratin K5Cre (From Jose L. Jorcano; Ramirez et al., 2004), Mouse: R26-H2B-eGFP (Abe et al., 2011), Mouse: Lama5 floxed and Lama5 null (Nguyen et al., 2005).
Mice were maintained in a 12h-light/12h-dark cycle and temperatures of 18–23C with 40–60% humidity.

Wild animals

Not used.

Field-collected samples

Not used.

Ethics oversight

All animal experiments were conducted and performed in accordance with approved Institutional Animal Care and Use Committee protocols in RIKEN Kobe Branch.

Note that full information on the approval of the study protocol must also be provided in the manuscript.

Flow Cytometry

Plots

Confirm that:

- [x] The axis labels state the marker and fluorochrome used (e.g. CD4-FITC).
- [x] The axis scales are clearly visible. Include numbers along axes only for bottom left plot of group (a 'group' is an analysis of identical markers).
- [x] All plots are contour plots with outliers or pseudocolor plots.
- [x] A numerical value for number of cells or percentage (with statistics) is provided.

Methodology

Sample preparation	Sample preparation procedures are also described in detail in Method. Dorsal skin tissues were surgically removed from 8-week-old mice and incubated for 1h with trypsin at 37C. Epithelial cells were collected from the tissue and filtered through a 40 um cell strainer to generate a single cell suspension. The remaining dermis tissues were minced with a scalpel and incubated with collagenase I for 2h at 37C. Cells were filtered through a 40 um cell strainer to generate a single cell suspension.
Instrument	FACSAria II
Software	FACSDiva 7 and 8.0 and FlowJo 9.9.3
Cell population abundance	Effectiveness of tissue separation and purity of the isolated cell pools were verified by the following qRT-PCR, RNA-seq and immunohistochemical analyses with cell compartment-specific markers. See FACS part of the Methods for detail.
Gating strategy	FACS gating strategy is shown in Supplemental Figure 1a-g and Methods. For the sorting of epithelial cells, to deplete haematopoietic and endothelial cells (lineage-positive cells), the cell suspension was stained with PE-Cy7-conjugated antibodies for CD45 (eBioscience, 30-F11), TER-119 (eBioscience, TER119) and CD31 (eBioscience, 390). The cell suspension was also stained with Sca-1-PerCP-Cy5.5 (eBioscience, D7), CD34-eFluor660 (eBioscience, RAM34), CD49f (alpha6 integrin)-PE (eBioscience, GoH3). To eliminate debris, a FSC/SSC gate was used. To further eliminate cell doublets, a FSC-H/FSC-A gate was used. Live (DAPI-negative) and Lineage-negative cells were collected. In Live/Lin- cell population, target epithelial cells were identified on the basis of eGFP and cell surface marker expressions and isolated with distinct gates. For the isolation of basal cells, CD49f+ cells were collected. For the isolation of lower isthmus cells, Sca1-/CD34- cells were identified and further examined for the expressions of Lgr6-eGFP and CD49f. Then, Sca1-/CD34-/Lgf6-eGFP+/CD49f+ cells were isolated as lower isthmus cells. For the isolation of upper bulge cells, Sca1-/CD34- cells were identified and further examined for the expressions of Gli1-eGFP and CD49f. Then, Sca1-/CD34-/Gli1-eGFP+/CD49f+ cells were isolated as upper bulge cells. For the isolation of mid-bulge cells, CD34+/CD49f+ cells were collected. For the isolation of hair germ cells, Sca1-/CD34- cells were identified and further examined for their expressions of Cdh3-eGFP and CD49f. Then, Sca1-/CD34-/Cdh3-eGFP+/CD49f+ cells were isolated as hair germ cells.<br><br>For the sorting of dermal cells, to deplete haematopoietic and endothelial cells (lineage-positive cells), the cell suspension was stained with PE-Cy7-conjugated antibodies for CD45 (eBioscience, 30-F11), TER-119 (eBioscience, TER119) and CD31 (eBioscience, 390). The cell suspension was also stained with CD49f-PE (eBioscience, GoH3) and CD34-eFluor660 (eBioscience, RAM34). To eliminate debris, a FSC/SSC gate was used. To further eliminate cell doublets, a FSC-H/FSC-A gate was used. Live (DAPI-negative) and Lineage-negative cells were collected. In Live/Lin- cell population, target dermal cells were identified on the basis of eGFP and cell surface marker expressions and isolated with distinct gates. For the isolation of dermal papilla cells, CD34-/Lef1-eGFPmid cells were identified and further examined for their CD49f expression. Then, CD34-/Lef1-eGFPmid/CD49f+ cells were collected as dermal papilla cells. For the isolation of pan-dermal fibroblasts, Pdgfra-eGFP+ cells were collected.

- [x] Tick this box to confirm that a figure exemplifying the gating strategy is provided in the Supplementary Information.
